# STAT3 promotes tumour progression in glioma by inducing FOXP1 transcription

**DOI:** 10.1111/jcmm.13837

**Published:** 2018-08-22

**Authors:** Xinlin Sun, Jihui Wang, Min Huang, Taoliang Chen, Jiansheng Chen, Fabing Zhang, Huijun Zeng, Zhimin Xu, Yiquan Ke

**Affiliations:** ^1^ National Key Clinical Specialty Guangdong Provincial Key Laboratory on Brain Function Repair and Regeneration Department of Neurosurgery Zhujiang Hospital Southern Medical University Guangzhou China; ^2^ Affiliated Bayi Brain Hospital PLA General Army Hospital Beijing China

**Keywords:** FOXP1, glioma, molecular mechanism, STAT3, transcription

## Abstract

**Objective:**

This paper investigated the effects of STAT3 through promoting FOXP1 transcription on proliferation, apoptosis and invasion in glioma cells.

**Methods:**

Quantitative real‐time PCR (qRT‐PCR) and Western blot assay were administered to assess the mRNA and protein expression levels of STAT3 and FOXP1 in glioma tissues and cells, respectively. Luciferase reporter and Chromatin Immunoprecipitation (ChIP) assays were implemented to determine the correlation between STAT3 and FOXP1. MTT and colony formation assays were conducted to identify cell growth. Flow cytometry was run to detect the cell apoptosis rate of glioma cells. Transwell assays were conducted to reveal cell invasion ability.

**Results:**

The mRNA and protein expression levels of STAT3 were highly expressed in glioma tissues and cells. After cells transfected with siRNA of STAT3, both STAT3 and FOXP1 were simultaneously downregulated. STAT3 directly regulated FOXP1 transcription. STAT3 promoted cell proliferation, inhibited cell apoptosis and enhanced cell invasion through promoting FOXP1 transcription in glioma cells.

**Conclusion:**

In summary, STAT3 gene was a transcriptional regulator of FOXP1. Depleted STAT3 restrained cell proliferation and invasion, promoted cell apoptosis in glioma cells. This molecular mechanism between STAT3 and FOXP1 can serve as a therapeutic target for glioma treatment.

## 1 INTRODUCTION

Glioma is one of the most prevalent intracranial malignancies and characterized as invasive growth without obvious boundaries distinguishing from adjacent brain tumour tissues.^1^ Despite the significantly enhanced diagnosis and therapy for glioma including excision, radiation therapy and chemical therapy, the prognosis of glioma remains unfavourable because of its cell proliferation and invasion into the adjacent normal brain parenchyma.^2^, ^3^, ^4^, ^5^, ^6^, ^7^ Furthermore, due to the invasive nature of high‐grade glioma, the 5‐year survival rates of high‐grade glioma patients are <10%.^8^ Although some studies have been carried out on the prognostic significance of glioma, it will be highly desirable to determine the molecular mechanisms for the glioma therapy that will be more effective for patients.^5^, ^9^

Signal Transducer and Activator of Transcription 3 (STAT3), a member of the STAT protein family, has played a vital role in transmitting signals form cytokines and growth factors.^10^, ^11^ The activation of the STAT3 protein is composed of a chain of events including dimerization of the N‐terminal domain, then Src Homology 2 (SH2) domain, the subsequent DNA‐binding domain and the final transcription activation of the C‐terminal domain.^12^, ^13^, ^14^ SH2 domain and the transcription activation are of great significance for STAT3 activation.^13^ However, STAT3 is constantly activated in a wide range of human malignant tumours including glioma.^15^, ^16^, ^17^, ^18^ And once activated, STAT3 is associated with oncogenesis and cancer progression through promoting the transcription of a few genes which control tumour cell viability, and inhibit cell apoptosis, vascularization and cell cycle.^19^, ^20^, ^21^, ^22^ To be more specific, the constitutively activated STAT3 elevates the expression of the downstream gene including Bcl‐2, Bcl‐xl, c‐myc, survivin and vascular endothelial growth factor (VEGF).^23^, ^24^, ^25^

Forkhead box protein P1 (FOXP1), a member of FOXP subfamily, is a transcriptional factor that has extensive functions.^26^ The FOXP subfamily is part of FOX proteins superfamily which has a highly preserved “fork‐head” DNA‐binding domain.^27^ FOX proteins mediate cell‐division cycle, cell viability, differentiation and apoptosis.^28^ FOXP1 serves as both potential oncogene and tumour suppressor due to its distinguishing expression levels in diverse tumour types.^29^ On the one hand, FOXP1 was reported to be an oncogene with overexpressed mRNA levels and protein levels of various human B‐cell lymphomas.^30^, ^31^, ^32^, ^33^, ^34^ On the other hand, silencing of FOXP1 has been associated with prostate cancer, renal cell cancer and breast cancer.^30^, ^31^, ^32^, ^35^, ^36^

Findings of the molecular mechanism of oncogenesis and progression of glioma are vital to ameliorate present treatments and develop new therapy. Up to now, however, the associations between STAT3 and FOXP1 expression in human glioma have not been examined. Our findings facilitate the perception of molecular mechanism and effects of STAT3 on glioma progression via inducing FOXP1 and usher in a novel therapy for gliomagenesis.

## 2 MATERIALS AND METHODS

### 2.1 Tissue samples and clinical characteristics

All glioma tissue samples were gained from a total of 71 patients undergoing operative treatment at the Zhujiang Hospital, Southern Medical University, after they signed the written informed consent. In total, 71 glioma tissues and 71 adjacent glioma tissues were stored frozen at −80°C until for the use of qPCR or western blot measurement. The current study was confirmed by the Ethics Committee of Zhujiang Hospital, Southern Medical University. Clinical characteristics were seen in Table 1.

**Table 1:** Characteristics of the 71 patients

Clinicopathologic parameters	N
Total cases	71
Gender	
Male	42
Female	29
Age	
≥60	39
<60	32
WHO grade	
Low(I‐II)	29
High(III‐IV)	42
Surgery	
Complete resection	38
Subtotal resection	33
Invasion of corpus callosum	
Yes	10
No	61
Location of cerebral hemisphere	
Left	34
Right	31
Bilateral	6
Movement disorder	
Yes	12
No	59

### 2.2 Cell culture

Normal human glial cells (SVG P12 and HA) and glioma cell lines (SHG‐44, U87, GOS‐3 and TJ905) were purchased from BeNa Culture Collection (Beijing, China).The SVG P12, HA, SHG‐44,U87 and TJ905 cell lines were maintained in Dulbecco's Modified Eagle's Medium (DMEM) with 4 mmol/L l‐glutamine and sodium pyruvate containing 10% fetal bovine serum (FBS) (Gibco; Thermo Fisher Scientific,Waltham, MA, USA). The GOS‐3 cells were cultivated in Minimum Essential Medium with Earle's Balanced Salts (MEM‐EBSS) including 4 mmol/L l‐glutamine and 10% FBS (Gibco; Thermo Fisher Scientific). Media were placed in incubators at 37°C with 5% CO_2_.

### 2.3 Cell transfection

The U87 and SHG‐44 cells transfection were conducted by Lipofectamine 2000 Transfection reagents (Invitrogen, Carlsbad, CA, USA) in accordance with manufacturer's protocol. Cells were cultured in 24‐well plates at 37°C with 5% CO_2_ until to a confluency of 70%‐80% and were transfected with siRNA of STAT3 (siRNA1 and siRNA2) or pcDNA3.1‐STAT3. For exploring the molecular mechanism of STAT3 and FOXP1, the U87 and SHG‐44 cells were also transfected with si‐NC plus pcDNA3.1 (NC), pcDNA3.1‐FOXP1 or siRNA1‐STAT3 plus pcDNA3.1‐FOXP1. SiRNA, pcDNA3.1‐STAT3 and pcDNA3.1‐FOXP1 vector were acquired from Shanghai GenePharma (Shanghai, China). These experiments were repeated twice.

### 2.4 qRT‐PCR

Total RNA was extracted by using the TRIzol^®^ Reagent (Invitrogen). After quantification by NanoDrop 2000 (Thermo Fisher Scientific Inc), 300 ng of sample RNA was reverse‐transcripted into cDNA using Omniscript RT kit (QIAGEN, Duesseldorf, Germany). Consequently, cDNA was analysed for PCR by THUNDERBIRD SYBR^®^ qPCR Mix (Toyobo, Japan). PCR conditions were conducted as follows: 10 minutes at 95°C followed by 45 cycles of 10 seconds at 95°C, 30 seconds at 60°C and 30 seconds at 40°C. The relative expression levels were analysed by 2^−ΔΔCT^ method and normalized to the expression of GAPDH. All reactions were run in triplicate. The primer sequences of STAT3, FOXP1 and GAPDH were as listed below: STAT3 forward: 5′‐AACTCTCACGGACGAGGAGCT‐3′, STAT3 reverse: 5′‐AGTAGTGAACTGGACGCCGG‐3′; FOXP1 forward: 5′‐TCCCGTGTCAGTGGCTATGAT‐3′, FOXP1 reverse: 5′CTCTTTAGGCTGTTTTCCAGCAT‐3′; GAPDH forward: 5′‐GAAGGTGAAGGTCGGAGTC‐3′, GAPDH reverse: 5′‐GAAGATGGTGATGGGATTTC‐3′.

### 2.5 Western blot

Total proteins were isolated from the cell lines. Quantitated by Pierce BCA Protein Assay Kit (Pierce, Rockford, IL, USA), up to 100 μg of total protein from each sample cell lysates were dissolved by SDS polyacrylamide gel electrophoresis (PAGE) and then moved to PVDF membrane. Subsequently, membranes were submerged in Tris Buffered Saline Tween (TBST) with 5% skim milk powder for 1 hours at room temperature and supplemented with primary antibodies (anti‐STAT3, ab119352, 1:5000; anti‐FOXP1, ab32010, 1:500; anti‐GAPDH, ab8245, 1:5000). The PVDF membranes were washed with TBST for three times and then added with secondary antibodies (Goat Anti‐Mouse IgG H&L, 1:3000). The whole antibodies were acquired from Abcam (Cambridge, MA, USA). The chromogenic reaction was visualized in all membranes by ECL Plus (GE Healthcare, Little Chalfont, Buckinghamshire, UK). Afterwards, integrated optical density of the protein bands were measured through Lab Works4.5 software (USA). GAPDH protein band is detected as internal control in each sample.

### 2.6 Luciferase reporter assays

For promoter analysis of the gene, sequences in promoter region were embedded into the upstream of luciferase reporter gene in pGL3 vector (Promega, Madison, WI, USA). Luciferase reporter assays were conducted in U87 and SHG‐44 cells. The cells were immersed in 24‐well dishes overnight and co‐transfected with luc‐FOXP1 containing firefly luciferase and si‐STAT3 or si‐NC. Then the cells were harvested 24 hours after transfection and renilla luciferase was conducted for normalization. Luciferase reporter assays were implemented by Dual Luciferase Reporter Assay system (Promega).

### 2.7 Chromatin immunoprecipitation assays

The chromatin immunoprecipitation (ChIP) assay kit was purchased through Upstate Biotechnology (Lake Placid, NY, USA). U87 or SHG‐44 cells were treated with formaldehyde to cross‐link protein and DNA to the FOXP1 promoter, and sonication was used to shear DNA. Chromatin was immunoprecipitated using antibodies against STAT3 or IgG isotype control (all from Abcam). IgG was applied as the negative control for the ChIP assay. The quantification of immunoprecipitated DNA was measured by qRT‐PCR. All data were normalized to the percentage of input.

### 2.8 MTT assay

Briefly, U87 or SHG‐44 cells were grown in 96‐well dishes at 37°C. Measurements were divided into four groups including NC group, siRNA1‐STAT3 group, pcDNA3.1‐FOXP1 and siRNA1‐STAT3 + p‐FOXP1 group. The cells growth was observed at 0, 24, 48 and 72 hours respectively. MTT solution was purchased through Beyotime Biotechnology (Shanghai, China). Cells were added with MTT kit at each time point and then added with DMSO. The absorbance was measured by enzyme immunoassay instrument at 570 nm.

### 2.9 Colony formation assays

In total, 1 × 10^3^ U87 or SHG‐44 cells were placed in six‐well plates and incubated at 37°C. After transfection, living cells were detected via trypan blue staining and calculated. A lower number of viable cells were then planted in 6‐well dishes and went on incubation for 2‐3 weeks. Subsequently, cells were rinsed with phosphate‐buffered saline (PBS) buffer and set with methanol. Crystal violet with methanol was used to stain transfected U87 cells for 10 minutes at room temperature. Finally, distilled water was used to wash and dry the plates. Each measurement was run in triplicate.

### 2.10 Flow cytometry

Cell apoptosis rate was caculated by flow cytometry assay. Experiments were randomly divided into NC group and transfected groups including siRNA1‐STAT3 group, pcDNA3.1‐FOXP1 and siRNA1‐STAT3 + p‐FOXP1 group. After collection, cells were rinsed and resuspended with PBS. Then Annexin V‐FITC/PI Apoptosis Detection Kit (BD Biosciences, San Jose, CA, USA) was used to stain cells as described in manufacturer's protocol. FACS Calibur (BD Biosciences) was run to determine the apoptosis rate of the stained cells. Data were measured by FACS Diva software. These experiments were repeated 3 times.

### 2.11 Invasion assays

Transwell units were used to conduct the cell invasion assays. The upper chamber membranes were coated with matrigel and the lower chamber was supplemented with FBS. After 48‐hour incubation, the remaining cells in the upper side were separated with cotton swabs, while the invaded cells were fixed in methanol for 30 minutes, stained with 0.1% crystal violet and counted under the observation of microscope. Theses assays were administered in triplicate. Mean ± SD value was considered as the cells invasion number.

### 2.12 Statistical analysis

The significance of the data between experimental groups was measured by Student *t* test, using GraphPad Prism 6.0 (GraphPad Prism; Version X; La Jolla, CA, USA). *P *< 0.05 was regarded as of statistical significance.

## 3 RESULTS

### 3.1 STAT3 was positively regulated in glioma tissues and cells

**Figure 1:**
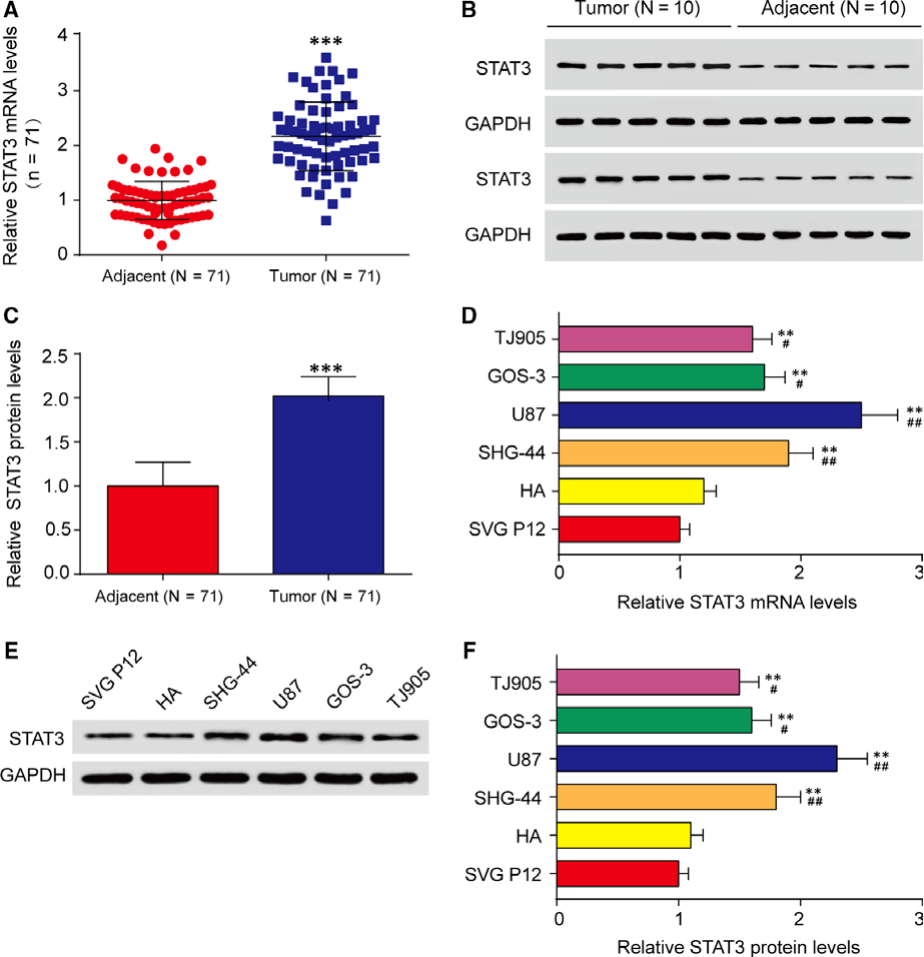
STAT3 was highly expressed in glioma tissues and cells. A, The STAT3 expression levels in adjacent and tumour glioma tissues were analysed by qRT‐PCR. ***P <* *0.001, compared with adjacent tissues. B‐C, The STAT3 protein levels in adjacent and tumour glioma tissues were analysed by western blot assay. GAPDH was regarded as an internal control. n = 10, ***P <* *0.001, compared with adjacent tissues. D, Relative STAT3 expression levels was detected in astrocyte cells (SVG P12 and HA) and glioma cell lines (SHG‐44, U87, GOS‐3 and TJ905) by qRT‐PCR. **P <* *0.01, compared with SVG P12; ^#^ *P *<* *0.05 and ^##^ *P *<* *0.01, compared with HA. (E‐F) Relative STAT3 protein levels were observed in SVG P12, HA, SHG‐44, U87 and GOS‐3 and TJ905 cell lines by western blot assay. **P <* *0.01, compared with SVG P12; ^#^ *P *<* *0.05 and ^##^ *P *<* *0.01, compared with HA

QRT‐PCR assay was implemented to measure STAT3 expression levels in tissue samples obtained from 71 para‐cancer tissues and 71 glioma tumour tissues. The result revealed that STAT3 was up‐regulated in glioma tissues (Figure 1A). Then the high protein levels of STAT3 in tumour tissues were determined by western blot assay compared with adjacent tissues (Figure 1B and C). QRT‐PCR demonstrated that STAT3 expression was dramatically increased in glioma cell lines (SHG‐44, U87, GOS‐3 and TJ905) compared with human astrocytic cells (SVG P12 and HA) (Figure 1D). Meanwhile, western blot analysis showed that STAT3 protein levels in glioma cell lines (SHG‐44, U87, GOS‐3 and TJ905) were higher than normal human astrocyte (SVG P12 and HA) (Figure 1E and F). In addition to that, among the human glioma cells including SHG‐44, U87, GOS‐3 and TJ905, U87 and SHG‐44 were with the highest and the second highest mRNA and protein levels of STAT3, respectively, which were selected as the main experimental subject.

### 3.2 STAT3 activated FOXP1 expression by binding to its promoter region

**Figure 2:**
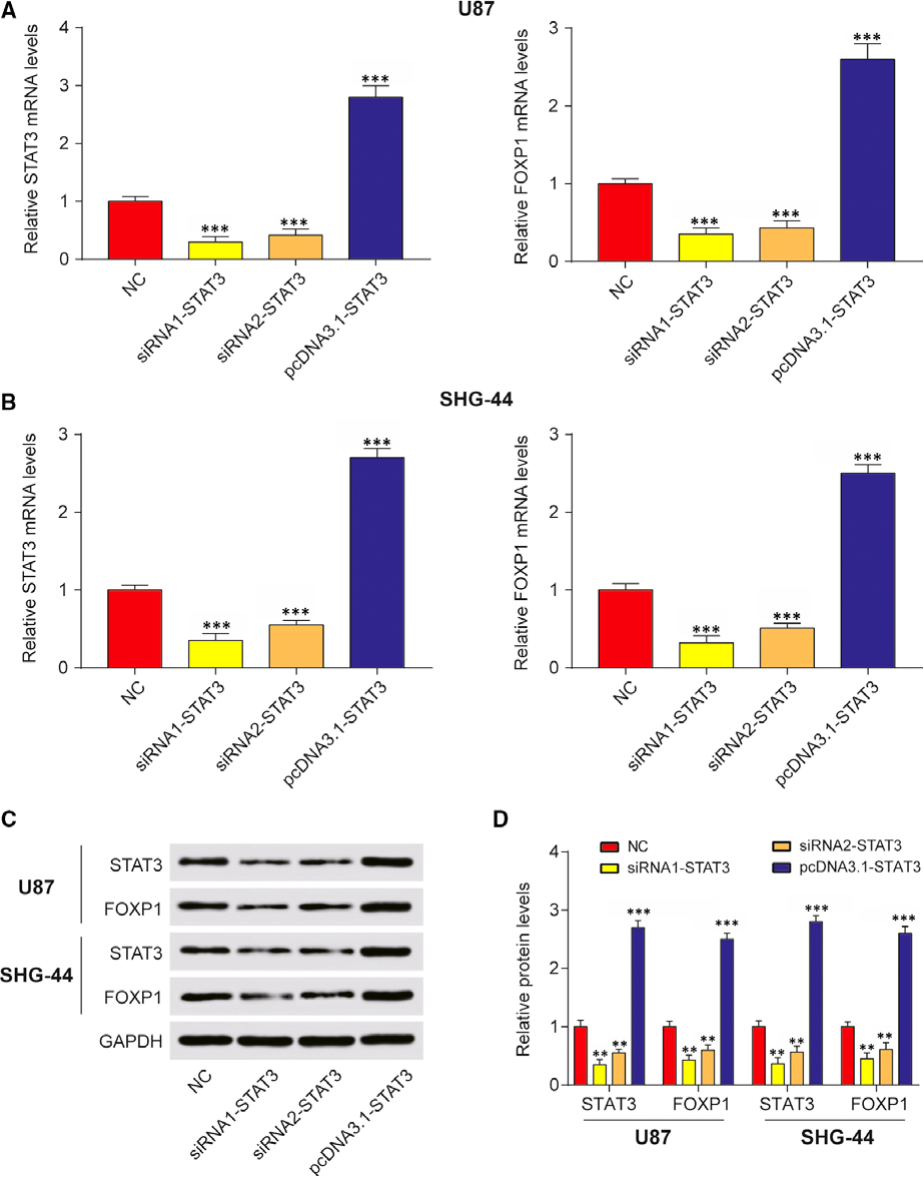
STAT3 directly regulated FOXP1 transcription. A, QRT‐PCR assay was implemented to determine the relative STAT3 and FOXP1 mRNA levels in U87 cells with NC, siRNA1‐STAT3, siRNA2‐STAT3 and pcDNA3.1‐STAT3 transfection. ***P <* *0.001 compared with NC. B, QRT‐PCR assay was implemented to determine the relative STAT3 and FOXP1 mRNA levels in SHG‐44 cells with NC, siRNA1‐STAT3, siRNA2‐STAT3 and pcDNA3.1‐STAT3 transfection. ***P <* *0.001, compared with NC. C‐D, Western blot assay was conducted to analyse relative STAT3 and FOXP1 protein levels in U87 and SHG‐44 cell with NC, siRNA1‐STAT3, siRNA2‐STAT3 and pcDNA3.1‐STAT3 transfection. **P <* *0.01, ***P <* *0.001, compared with NC

**Figure 3:**
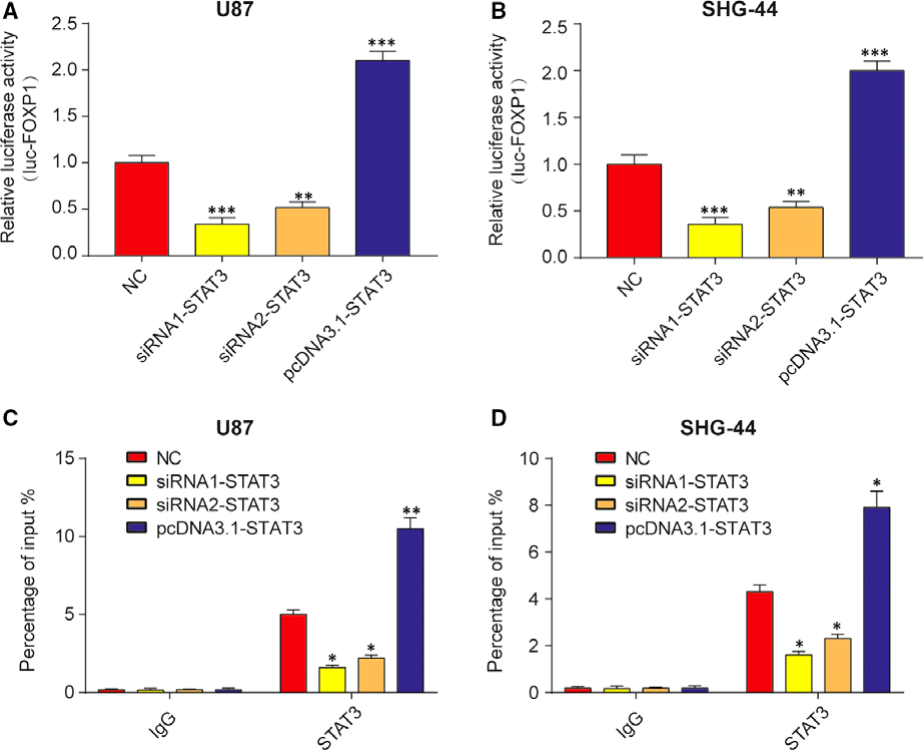
STAT3 directly regulated FOXP1 transcription. A‐B, Down‐regulation of STAT3 inhibited luciferase activity of luc‐FOXP1 and up‐regulation of STAT3 promoted luciferase activity of luc‐FOXP1 in U87 and SHG‐44 cells. **P <* *0.01, ***P <* *0.001, compared with NC. C‐D, ChIP assay was used to analyse the binding site of STAT3 in FOXP1 promoter in U87 and SHG‐44 cells. Results were counted from percentage of input. *P <* *0.05, **P <* *0.01, compared with NC

Some reports documented that FOXP1 was closely correlated with human glioma progression.^37^ However, the association between STAT3 and FOXP1 has not yet been made clear. Thus, we next examined whether STAT3 could be a transcriptional promoter of the FOXP1 gene. At the beginning, U87 and SHG‐44 cells were transfected with siRNA1‐STAT3, siRNA2‐STAT3 or pcDNA3.1‐STAT3. Then qRT‐PCR analysis revealed that mRNA levels of STAT3 and FOXP1 were simultaneously down‐regulated in groups of siRNA1‐STAT3 and siRNA2‐STAT3 compared with NC group (Figure 2A and B). Western blot analysis revealed that the protein levels of STAT3 and FOXP1 were sharply decreased in siRNA transfected U87 or SHG‐44 cells compared with NC cells (Figure 2C and D). Thus, down‐regulation of STAT3 inhibited mRNA and protein levels of FOXP1. To test the hypothesis that STAT3 can directly regulate FOXP1 transcription, luciferase reporter assays and chromatin immunoprecipitation (ChIP) assays were implemented. Luciferase reporter assays demonstrated the stimulative effect of STAT3 on FOXP1 promoter in U87 and SHG‐44 cells after transfection for 48 hours. The data revealed that, compared with NC group, the knockdown of STAT3 decreased luciferase activity of FOXP1, while overexpression of STAT3 increased luciferase activity (Figure 3A and B). Next ChIP assays were conducted to analyse whether STAT3 directly bonded the FOXP1 promoter. As expected, STAT3 protein bound the FOXP1 promoter was dramatically increased in U87 and SHG‐44 cells. In other words, binding of STAT3 to the FOXP1 promoter enhanced transcription of FOXP1 (Figure 3C and D). Besides, the inhibition effects of siRNA1‐STAT3 were stronger than siRNA2‐STAT3. Therefore, siRNA1‐STAT3 was selected to continue the next experiments. In short, STAT3 was the transcriptional regulator of FOXP1.

### 3.3 STAT3 promoted glioma cell proliferation through promoting FOXP1 transcription

**Figure 4:**
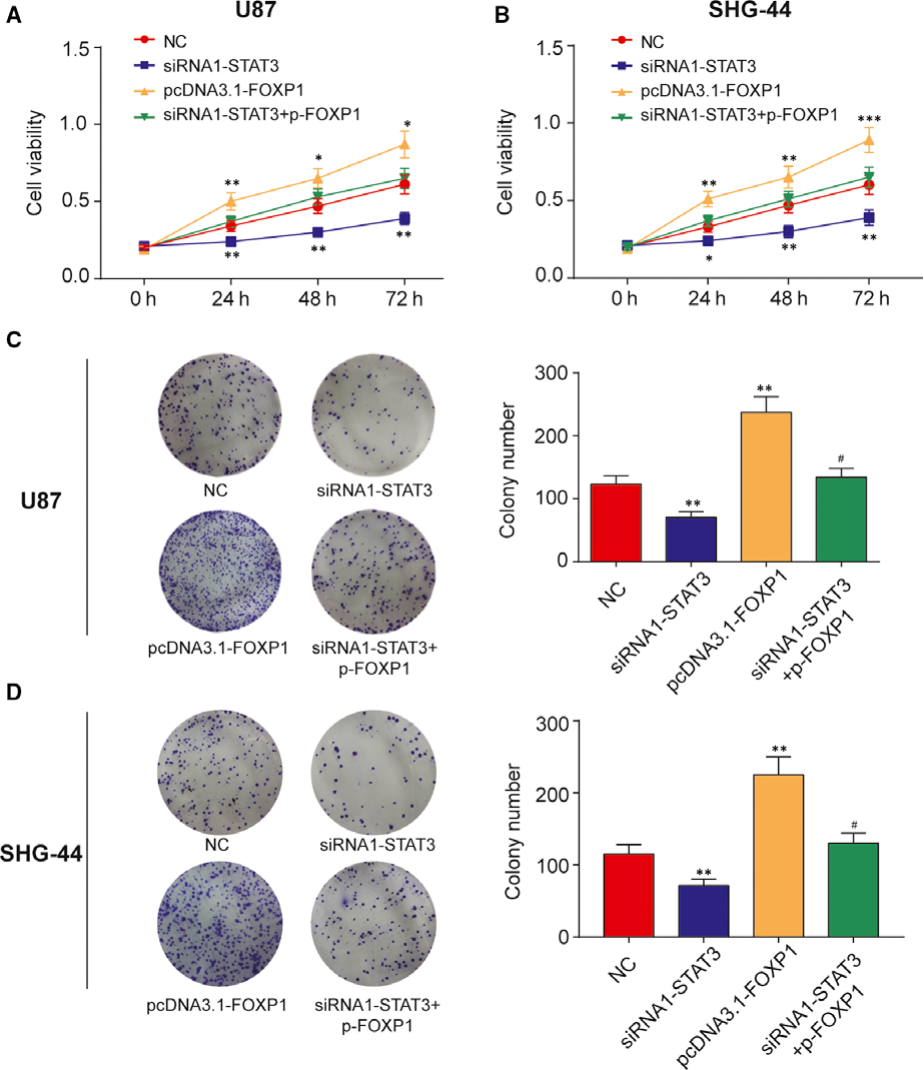
The silencing of STAT3 inhibited cell proliferation by inhibiting FOXP1 transcription. A‐B, MTT assay was conducted to determine the cell viability after transfected with siRNA1‐STAT3, pcDNA3.1‐FOXP1, siRNA1‐STAT3 plus pcDNA3.1‐FOXP1 or negative control (NC) at 0, 24, 48 and 72 hours in U87 and SHG‐44 cells. *P <* *0.05, **P <* *0.01, compared with NC. C‐D, Cell proliferation also was measured by the colony formation assays after transfected with siRNA1‐STAT3, pcDNA3.1‐FOXP1, siRNA1‐STAT3 plus pcDNA3.1‐FOXP1 or negative control (NC). **P <* *0.01, compared with NC. ^#^ *P *<* *0.05, compared with siRNA1‐STAT3 or pcDNA3.1‐FOXP1

To determine whether STAT3 promoted glioma progression via FOXP1 induction, we performed rescue experiment in U87 and SHG‐44 cells. At first, U87 or SHG‐44 cell lines were transfected with NC, siRNA1‐STAT3, pcDNA3.1‐FOXP1, siRNA1‐STAT3 plus pcDNA3.1‐FOXP1, subsequently measured for cell proliferation by MTT and colony formation assays. Then, as measured by MTT assay, the knockdown of STAT3 suppressed cell growth while the elevation of FOXP1 enhanced cell viability in U87 and SHG‐44 cells compared with NC group (Figure 4A and B). Additionally, reducing glioma cell viability dramatically induced by STAT3 inhibition was rescued via FOXP1 upregulation. The colony number of U87 and SHG‐44 cells was markedly decreased after STAT3 knockdown and was dramatically increased after FOXP1 upregulation, while FOXP1 restoration abolished the effect of STAT3 knockdown (Figure 4C and D). Therefore, MTT and colony formation assays revealed that STAT3 depletion inhibited cell proliferation via inhibiting FOXP1 transcription.

### 3.4 Down‐expression of STAT3 promoted cell apoptosis by reducing FOXP1 transcription

**Figure 5:**
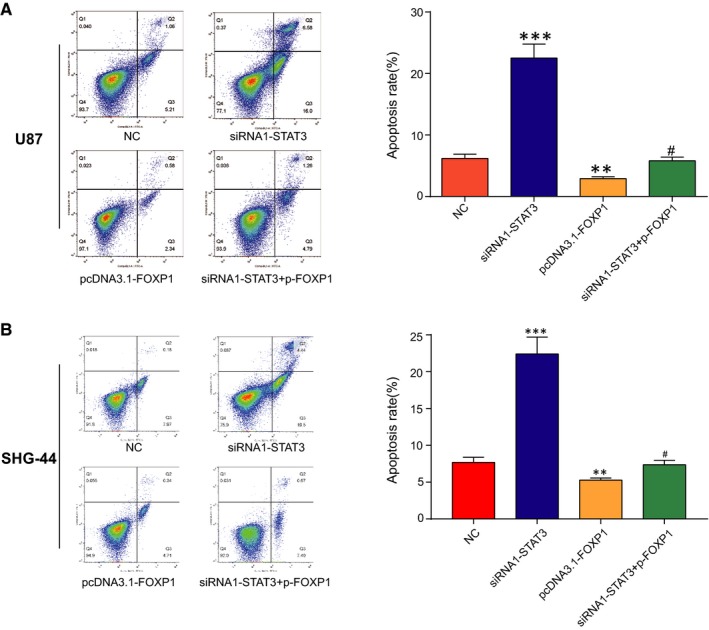
The silencing of STAT3 promoted cell apoptosis through inhibiting FOXP1 transcription. A‐B, Double staining for annexin V/PI was used to detect the U87 and SHG‐44 cells apoptosis rate by flow cytometry after transfected with siRNA1‐STAT3, pcDNA3.1‐FOXP1, siRNA1‐STAT3 plus pcDNA3.1‐FOXP1 or negative control (NC). **P <* *0.01, compared with NC. ^#^ *P *<* *0.05, compared with siRNA1‐STAT3 or pcDNA3.1‐FOXP1

Furthermore, flow cytometry assay was applied to analyse the apoptosis rate of U87 and SHG‐44 cells transfected with siRNA1‐STAT3, pcDNA3.1‐FOXP1, siRNA1‐STAT3 plus pcDNA3.1‐FOXP1 or negative control (NC). The results demonstrated that the apoptosis rate of siRNA1‐STAT3 group soared while that of pcDNA3.1‐FOXP1 group dramatically fell compared with NC group. And up‐regulation of FOXP1 rescued the increasing apoptosis rate caused by knocking‐down of the STAT3 (Figure 5A and B). In other words, down‐regulated STAT3 expedited the cell apoptosis, but elevated FOXP1 inhibited the cell apoptosis. Thus, down‐expression STAT3 promoted cell apoptosis by suppressing FOXP1 transcription.

### 3.5 STAT3 promoted cell invasion by inducing FOXP1 transcription

**Figure 6:**
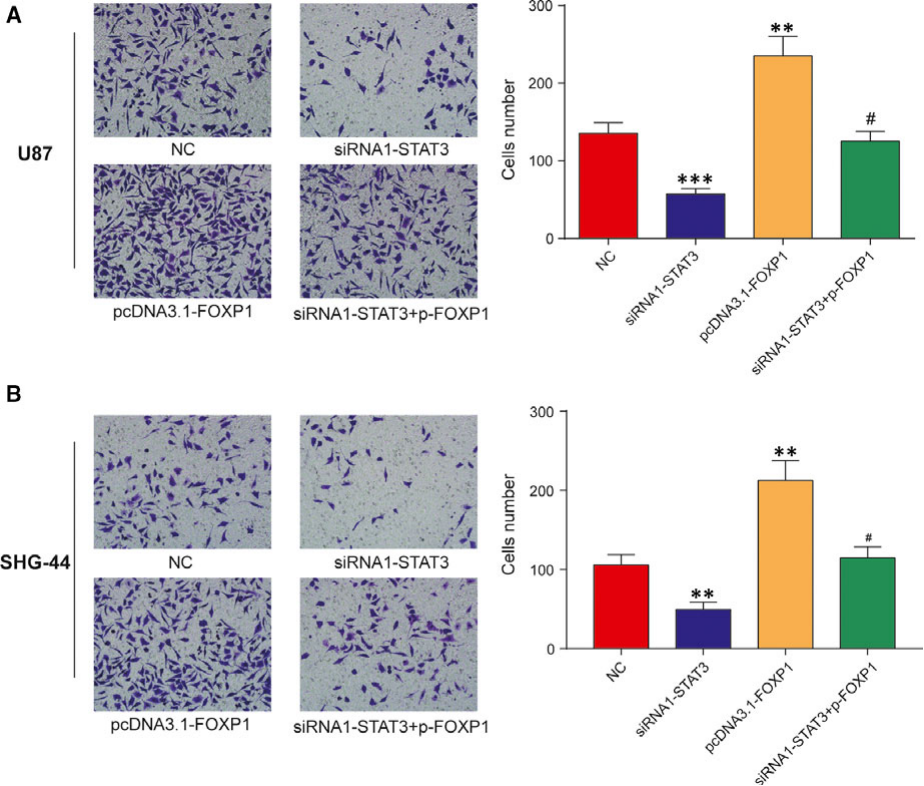
The silencing of STAT3 inhibited U87 and SHG‐44 cells invasion by reducing FOXP1 expression. A‐B, Representative invasion images and the relative number of invasive cells after transfected with siRNA1‐STAT3, pcDNA3.1‐FOXP1, siRNA1‐STAT3 plus pcDNA3.1‐FOXP1 or negative control (NC) in U87 and SHG‐44 cells. **P <* *0.01, ***P <* *0.001, compared with NC. ^#^ *P *<* *0.05, compared with siRNA1‐STAT3 or pcDNA3.1‐FOXP1

Finally, transwell invasion assays were employed to examine whether the effect of depleted STAT3 on U87 and SHG‐44 cells invasion ability could be restored by overexpression of FOXP1. Our observations demonstrated that the silencing of STAT3 gene lessened invaded cells number, while the elevation of FOXP1 dramatically increased invaded cells number compared with NC group (Figure 6A and B). In addition to that, upregulation of FOXP1 rescued the inhibition of cell invasion after STAT3 knockdown, which led to a dramatic rise of invaded cells number in siRNA1‐STAT3 plus pcDNA3.1‐FOXP1 group compared with siRNA1‐STAT3 group. In other words, down‐regulated STAT3 inhibited the invasive ability of U87 and SHG‐44 cells, which was rescued by upregulated FOXP1. Therefore, STAT3 enhanced cell invasive ability by inducing FOXP1.

## 4 DISCUSSION

Related reports had revealed that STAT3 expressions were associated with prognosis of diverse types of tumours such as breast cancer, acute myelogenous leukaemia and glioma, among others.^38^, ^39^, ^40^ Previously, a few studies had reported the overexpression of STAT3 in certain cancer types including intrahepatic cholangiocarcinoma, breast cancer and high‐grade glioma.^18^, ^41^, ^42^ Additionally, Carro et al^43^ reported a relationship between STAT3 expression and the mesenchymal transformation of glioma, suggesting that STAT3 was a “master regulator” of glioma transformation. In view of this, we hypothesized that STAT3 overexpression is associated with glioma. As expected, our findings demonstrated that STAT3 expression levels were elevated at both mRNA and protein levels in human glioma tissues and cells.

The molecular mechanisms of STAT3 regulation in glioma remain elusive. To determine the mechanism by which STAT3 regulates glioma progression, we predicted its target genes according to a series of related reports. Subsequently, FOXP1 was revealed to be closely connected with glioma oncogenesis and progression with proliferation, migration and invasion suppression of glioma cells.^44^ Meanwhile, Cui et al^44^ suggested that miR‐504 suppressed cell proliferation and induced apoptosis by downregulating FOXP1 in human glioma. What's more, FOXP1 was reported by Tian et al^45^ to be an oncogene by increasing cell growth in glioma cells. Gomez et al^46^ also held that downregulation of miR‐9 upregulated FOXP1 to promote tumorigenicity in glioma cells. These reports indicated that FOXP1 was an oncogene in glioma cells, which were similar to our findings. However, less report has dealt with the direct correlation of STAT3 and FOXP1 in glioma cells. Given that, STAT3 expression was depleted by transfection with small interfering RNA (siRNA) in glioma cells, in an attempt to elaborate whether STAT3 has a certain bearing on FOXP1 in glioma cells. Luciferase reporter assays and ChIP assays demonstrated that STAT3 is a direct regulator of the FOXP1 transcription. This was the first time that the molecular mechanism between STAT3 and FOXP1 in glioma cells was discussed.

Furthermore, in our rescue experiments, we explored whether the downregulation of STAT3 alters the biological function of glioma cells by inducing FOXP1. According to Konnikova et al^47^, the depleted STAT3 inhibited cell proliferation and induced cell apoptosis in glioma cells. Chen et al^48^ also reported that silencing of STAT3 decreased invasion activity and induced apoptosis of human glioma cells. These findings were similar to our results that STAT3 drove proliferation and invasion, and suppressed cell apoptosis of glioma cells by promoting FOXP1 transcription.

The current study featured the latent molecular mechanism of STAT3 in glioma cells. Firstly, we manifested the high expression levels of STAT3 in glioma tissues and cell lines. Next, we perceived there was a positive correlation between STAT3 and FOXP1. Finally, experimental results demonstrated that STAT3 knockdown restrained cell proliferation and invasion, and expedited cell apoptosis by reducing FOXP1. In a word, these results yielded that STAT3 and FOXP1 may be underlying therapeutics for glioma.

## CONFLICT OF INTEREST

The authors confirm that there are no conflicts of interest.
